# Symmetric and Asymmetric Semi-Metallic Gasket Cores and Their Effect on the Tightness Level of the Bolted Flange Joint

**DOI:** 10.3390/ma18112624

**Published:** 2025-06-04

**Authors:** Przemysław Jaszak, Rafał Grzejda

**Affiliations:** 1 Faculty of Mechanical and Power Engineering, Wroclaw University of Science Technology, Wybrzeze Stanislawa Wyspianskiego St. 27, 50-370 Wroclaw, Poland; 2 Faculty of Mechanical Engineering and Mechatronics, West Pomeranian University of Technology in Szczecin, 19 Piastow Ave., 70-310 Szczecin, Poland

**Keywords:** semi-metallic gasket, tightness level, bolted flange joint

## Abstract

The paper presents the effect of the symmetric and asymmetric semi-metallic gasket core shape on the tightness level in bolted flange joints. Experimental tests, as well as numerical calculations based on the finite element method, revealed that the asymmetric gasket core provides a higher strain on the sealing graphite layer and leads to a more uniform distribution of strain on the particular ridges of the core. Furthermore, the leakage rate of the asymmetric gasket was reduced by approximately 60% compared to the symmetric gasket. It was also observed that the uniformity of pressure and strain distribution in a gasket with an asymmetric core occurs over about 80% of the gasket width. The leakage reduction effect in a flange joint sealed with a gasket with an asymmetric core was theoretically explained. As shown, the main leakage flows through the porous structure of the graphite layer, while the leakage path at the interface between the metal rough profile and the graphite layer is several orders of magnitude smaller.

## 1. Introduction

Bolted joints are one of the most commonly used solutions for connecting flanges in piping systems [1,2,3,4]. Both symmetric and asymmetric fastener arrangements are used in these joints. The asymmetric geometry combined with the asymmetric fastener arrangement usually results in an uneven loading condition of the joint.

Non-uniformity in loading on parts of machines and equipment is an undesirable phenomenon, influencing reduced durability or unexpected destruction. One of the main design problems in this case is to minimise, and equalise, the load in the form of forces, moments and their derivatives, i.e., stresses [5,6,7]. An asymmetric load condition significantly reduces the fatigue life of machine parts, as exemplified by the results of numerous papers on the subject [8,9]. The levelling or reduction in the stress distribution can be realised via the so-called flexibility of the structure in regions of high stress concentration.

A typical device that is subjected to an uneven load condition is a bolted flange joint with a gasket [10,11,12]. As it turns out, all its components are subjected to uneven loading [13]. Flanges are subjected to bending as a result of bolt tension [14]. The deformation of the flange due to bending induces the bending of the bolt and also causes uneven pressure distribution on the contact surface of the gasket [15]. The effects of excessive flange deformation on the load condition of the joint and the level of gasket leakage are presented in [16,17]. Exceeding the permissible contact stress of a gasket can lead, for example, to the buckling of the inner coils—as occurs in a spiral gasket [18,19,20]. There are many examples of papers describing experimental tests [21,22,23,24,25] or numerical tests [26,27,28,29,30] of gasketed bolted flange joints. A review of these provides an insight into the phenomena occurring during non-uniform loading of these joints.

One way to equalise the pressure distribution on the gasket surface is to choose the right elasticity. The most commonly used type of gaskets, which have developed quite strongly in recent times, is semi-metallic gaskets [31]. They constitute a group of so-called high-performance gaskets, used at high pressure and temperature. Their characteristic feature is a construction consisting partly of metal and the remaining part of a soft material, the so-called filler [32,33]. The metal part can be the internal reinforcement of the gasket, e.g., in the form of a metal mesh, tape or sheet covered with a filler material. In other solutions, the metal part may be on the outer surface of the soft material, e.g., by fitting it completely. In the latter case, the soft material gives the gasket more flexibility while the metal part performs the sealing function. One of the most common solutions for a semi-metallic gasket is the multi-edge gasket. Its construction consists of a metal core (usually made of acid-resistant steel), whose serrated faces are covered with a filling material [34,35]. The filling material can be expanded graphite [36]. The advantages of this solution were presented in [34], analysing the effect of the shape of the gasket core on the level of leakage. As it turns out, making the core more flexible by undercutting its edges has a significant impact on the distribution of contact pressure that prevails at the faces.

The aim of the present study, in contrast to the results presented in [34,35], is to estimate the leakage at relatively low contact pressure. According to research [35], the strain of the graphite layer above a certain value fixes its permeability at a constant level. Below this value, the permeability is strongly dependent on the local strain value of the sealing layer. In the present study, an attempt was made to determine, analytically and numerically, the leakage at a non-uniform pressure distribution of the sealing graphite layer, for which the permeability depends on the local strain value. A model of fluid flow through a ring-shaped porous bed was adapted for this purpose.

On the basis of the tests carried out, it was shown that the asymmetric gasket core provides greater strain on the sealing graphite layer and leads to a more uniform distribution of strain on the individual ridges of the core. In addition, the leakage reduction effect in a flange joint sealed with a gasket with an asymmetric core was theoretically explained. It was also shown that the main leakage flows through the porous structure of the graphite layer, while the leakage path at the interface between the metal rough profile and the graphite layer is several orders of magnitude smaller.

## 2. Materials and Methods

### 2.1. Research Object

**Figure 1:**
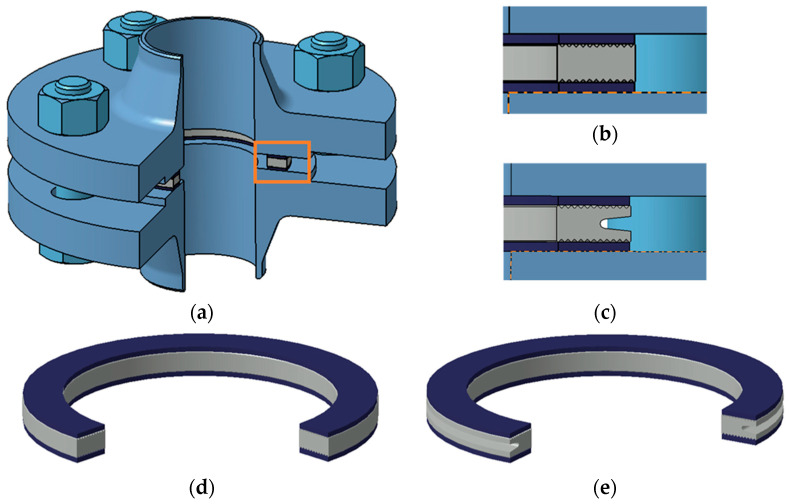
Components of the bolted flange joint: (**a**) view of the joint; (**b**) gasket section with symmetric core; (**c**) gasket section with asymmetric core; (**d**) view of the gasket with symmetric core; (**e**) view of the gasket with asymmetric core.

The object of the research was a multi-edge gasket made in two variants that differed in the shape of the cross-section of the metal core. The first variant had a core with a standard symmetric (rectangular) shape, while the second variant was characterised by an asymmetric cross-sectional shape that had a circumferential notch on the outer diameter of the core. Both variants are shown in Figure 1.

The advantages of a gasket solution with an undercut core were presented, among others, in [34], in which it was shown that such a solution significantly improves the tightness of the bolted flange joint.

The metal core was made from 316L steel, while the caps were made from expanded graphite with a thickness of 1 mm and a density of 1 g/cm^3^. The main overall dimensions of both structures (inner and outer diameter and thickness, as well as the shape and number of core edges cut into the faces) followed directly from EN 1514-6 [37,38].

### 2.2. Numerical Calculations

The objective of the numerical calculations was to determine the pressure and strain distributions on the surface of the graphite sealing layer, which deforms unevenly when in contact with the multi-edged surface of the metal core. The results of the calculations, mainly in the form of averaged strain values of the graphite layer on the individual ridges of the metal core, provided the necessary data for estimating the leakage value in an analytical manner. The calculations were performed in Ansys Workbench 19.1 (Ansys, Inc., Southpointe 2600 Ansys Drive, Cecil Township, PA 15317, USA) using structural static analysis [39,40].

#### 2.2.1. Calculation Model

**Figure 2:**
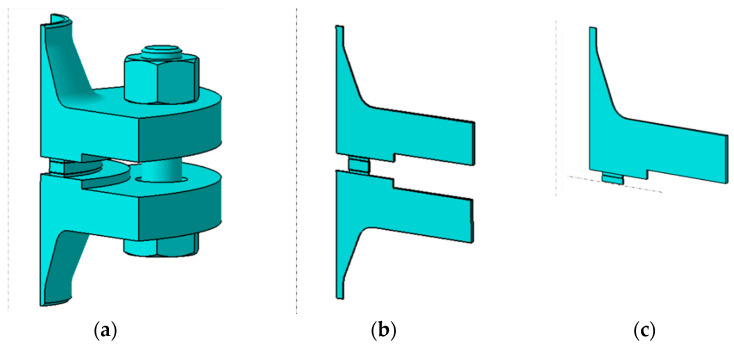
Model of the joint: (**a**) geometry simplified to 1/4 of the actual model; (**b**) geometry simplified to 1/360 of the actual model; (**c**) geometry simplified to 1/360 of the actual model and using symmetry in the gasket centre plane.

The computational model was a fragment of a DN40 PN40 bolted flange joint [41,42] with a multicore gasket in two configurations, i.e., with the symmetric and asymmetric core. In order to speed up the calculations, the geometric model was simplified to a joint fragment reflecting its cyclicity with respect to the axis of rotation and using symmetry in the horizontal split plane of the gasket [43,44,45,46]. The adopted model in the form of a three-dimensional joint slice with an angle of 1° is shown in Figure 2. This calculation approach is fully acceptable and approved by the Pressure Vessel Research Council (PVRC), as presented in [47]. The model in this form has no bolt geometry the same approach used in [48]. The load on the joint and the gasket, resulting from bolt tension, is in this case induced via the displacement applied at the point of contact between the bolt head (or nut) and the flange. The target value of bolt tension is measured using the reaction force induced via the displacement of the flange on the restrained part of the gasket core.

**Figure 3:**
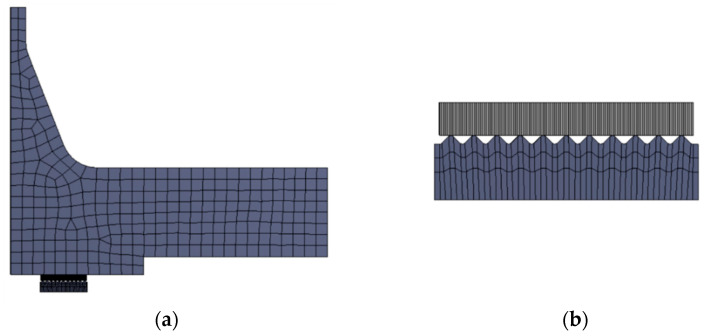
Discrete model of the joint: (**a**) view of the full finite element mesh; (**b**) finite element mesh in the vicinity of the gasket core and sealing layer.

SOLID186 higher-order shape function elements [49,50] were used to discretise the model. The finite element mesh is shown in Figure 3. The basic finite element size in the flange geometry area was 2 mm. For the metal core and graphite overlays, the element edge size was 0.01 mm and 0.05 mm, respectively.

**Figure 4:**
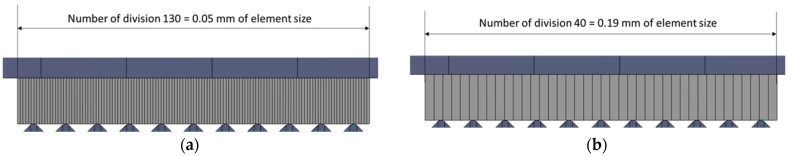
Mesh-independent test of the graphite layer: (**a**) mesh view with an element size of 0.05 mm; (**b**) mesh view with an element size of 0.19 mm.

**Figure 5:**
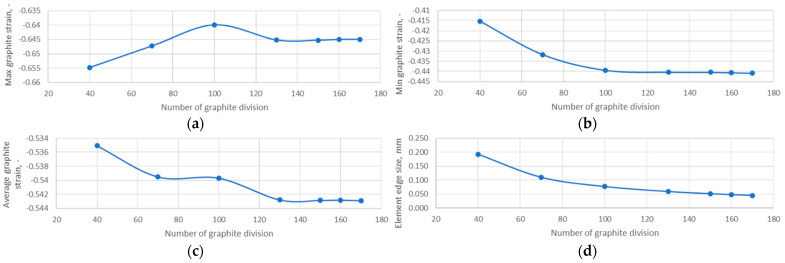
Results of the mesh-independent test of the graphite layer: (**a**) maximum graphite strain; (**b**) minimum graphite strain; (**c**) average graphite strain; (**d**) element edge size.

To investigate the effect of the grid size used to discretise the graphite layer on its strain values, a mesh-independent test was performed. The results of these calculations are shown in Figure 4 and Figure 5.

**Figure 6:**
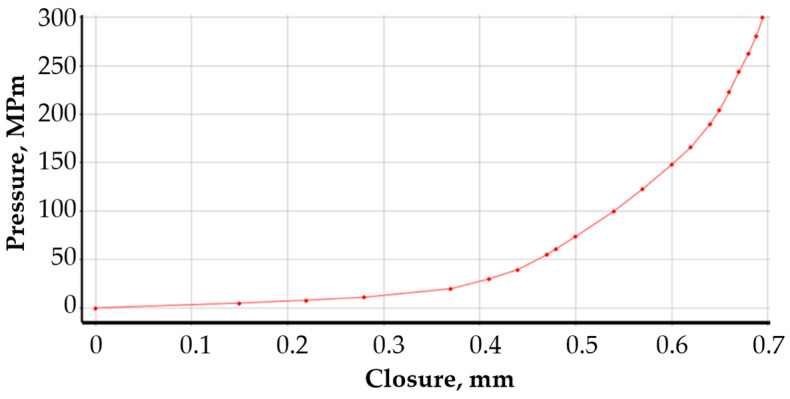
Compressive characteristics of a 1 mm-thick graphite sealing layer.

An elastic model was used to represent the mechanical properties of the flange and the gasket core, assuming a value of Young’s modulus and Poisson’s ratio. The material properties of the metal part of the joint were collected in Table 1. To represent the properties of the graphite caps, a non-linear gasket-type model available in the material database of the calculation programme used was applied. The compression characteristics of the graphite introduced in this material model were obtained from tests (based on EN 13555 [51,52]). The course of this characteristic is shown in Figure 6.

#### 2.2.2. Boundary Conditions

**Figure 7:**
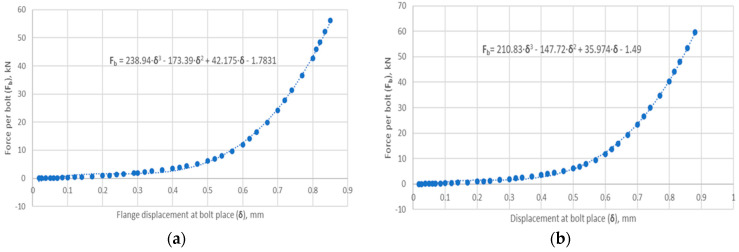
The relationship between the bolt force (as one-quarter of the reaction force) and flange displacement for (**a**) joint with symmetric gasket and (**b**) joint with asymmetric gasket.

The model was restrained in the horizontal plane of symmetry of the core division. A rotation constraint about the axis and a displacement constraint in the radial direction were applied to the cylindrical part of the flange neck. At the point of contact between the bolt head and the flange, a displacement was introduced to simulate its deformation resulting from the gradual tightening of the nut. This simulated the tension of the bolt. The specific values of the flange displacement applied at the bolt location were determined by measuring the reaction forces on the fixed support of the model. The total reaction force is the sum of the four bolt tensions. The relationship between the bolt force (as one-quarter of the reaction force) and flange displacement (in the bolt area) is shown in Figure 7.

**Figure 8:**
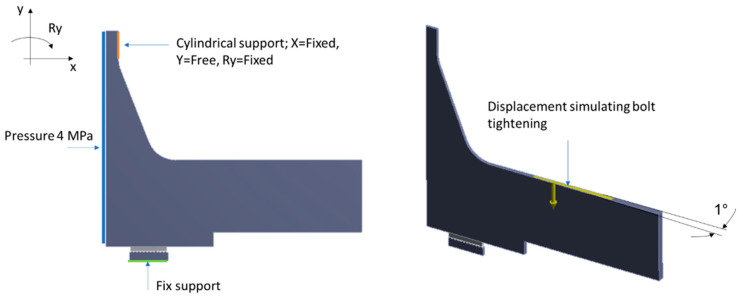
Boundary conditions of the calculation model.

A pressure of 4 MPa was applied to the inner walls of the model from the pressure of the medium to be sealed. The maximum displacement value was 0.8 mm. The target tension for a single bolt was 25 kN. The boundary conditions are presented in Figure 8. The contact between the graphite surface and the metal surfaces of the flange and the sealing core was defined as frictional, with the graphite surface set as ‘Contact’ and the metal surfaces as ‘Target’. The discretisation parameters of the contact surfaces are presented in Table 2.

### 2.3. Analytical Calculations

**Figure 9:**
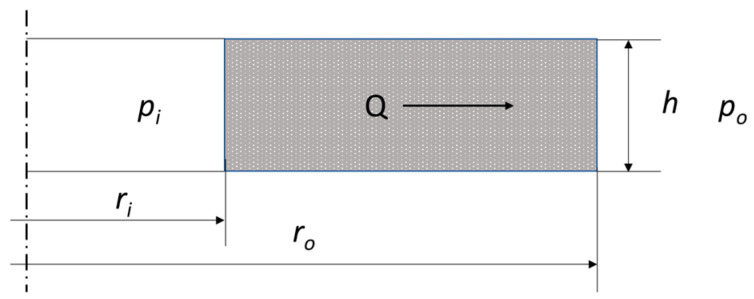
Geometric model of fluid flow through a porous bed in the form of a cylinder.

The objective of the analytical calculations was to estimate the leakage through the graphite caps of the analysed sealing solutions in two variants of the shape of the metal core. A model of fluid flow through a cylindrical porous bed, shown in Figure 9, was adopted as the analytical model.

The equation linking the basic geometrical data of the porous bed, and the properties of the medium are described in the following equation:

(1) $$Q=\frac{2\pi hK_{V}(p_{o}-p_{i})}{\mu ln\left(\frac{r_{o}}{r_{i}}\right)}$$

where *h* denotes the bed thickness, *K_V_* denotes the bed permeability, *p_i_* and *p_o_* denote the pressure inside and outside of the cylinder, respectively, *μ* denotes the dynamic fluid viscosity, and *r_i_* and *r_o_* denote the inner and outer radius of the cylinder.

Equation (1) allows the flow through a porous gasket layer to be modelled when the contact pressure on the gasket surface is uniform. Otherwise, the thickness of the layer (cap) and its permeability depends on its degree of deformation (compression). When the contact pressure on the sealing surface is uneven, it seems appropriate to isolate the zones (sealing widths) in which there is quasi-uniform pressure, calculate the permeability and layer thickness in these zones, and then add up the resulting partial values. In the case of the structure shown in Figure 1, the pressure concentration zones of the graphite layer are caused by the individual ridges cut into the metal core of the gasket. By transforming Equation (1), it is possible to count the pressure drop that will occur as a result of fluid flow through a single zone of pressure concentration of the graphite layer, caused by a given ridge of the gasket with known inner and outer radii, *r_i_* and *r_i_*_+1_:

(2) $$∆p=\frac{Q\mu ln\left(\frac{r_{i+1}}{r_{i}}\right)}{2\pi hK_{V}}$$

The total pressure drop across the individual pressure-back zones, Δ*p*, must be equal to the total pressure drop that occurs between the inner and outer surfaces of the graphite layer:

(3) $$∆p=∆p_{i}+∆p_{i+1}+\cdots +∆p_{n}$$

where Δ*p_i_* denotes the pressure drop of the fluid in the *i*-th zone of contact pressure concentration of the graphite layer, Δ*p_i_*_+1_ denotes the pressure drop of the fluid in the next adjacent zone of pressure concentration of the graphite layer, and Δ*p_n_* denotes the pressure drop of the fluid in the *n*-th (last) zone of contact pressure concentration of the graphite layer.

As the fluid flow through the layer is constant, the pressure difference across the outer and inner diameters is known, so the fluid flow through the layer with uneven contact pressure distribution can be calculated from the following equation:

(4) $$Q=\frac{2\pi (p_{o}-p_{i})}{\mu }\cdot \sum_{i=1}^{n}\left(\frac{h_{i}K_{Vi}}{ln\left(\frac{r_{i+1}}{r_{i}}\right)}\right)$$

**Figure 10:**
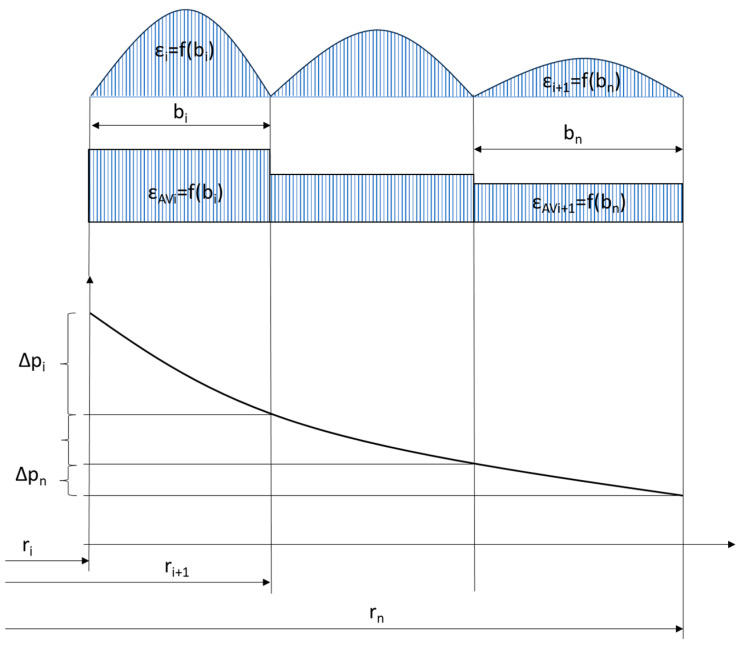
Graphical interpretation of the calculation of the partial strain and permeability values of the graphite sealing layer in the different concentration zones.

In addition, it can be assumed that at each of the ridges of the metal core, the strain and pressure of the graphite sealing layer were brought to an average value. A graphical interpretation of Equation (4) is shown in Figure 10.

As the thickness of the graphite layer in each zone is strain-dependent, the following can be written:

(5) $$h=h_{0}(1-\varepsilon )$$

where *h*_0_ denotes the nominal thickness of the graphite layer, and *ε* denotes the strain of the graphite layer.

Furthermore, when the fact that there are two graphite layers in the gasket is taken into account, the density of the fluid is known, and the leakage is related to the average circumference of the gasket, conclusively, Equation (4) can be written in the following form:

(6) $$Q=\frac{2h_{0}(p_{o}-p_{i})\rho }{\mu r_{AV}}\cdot \sum_{i=1}^{n}\left(\frac{\left(1-\varepsilon_{i}\right)K_{Vi}}{ln\left(\frac{r_{i+1}}{r_{i}}\right)}\right)$$

where *ρ* denotes the density of the fluid to be sealed, *ε_i_* denotes the strain of the *i*-th zone of the graphite layer, and *r_AV_* denotes the average gasket radius.

### 2.4. Experimental Research

**Figure 11:**
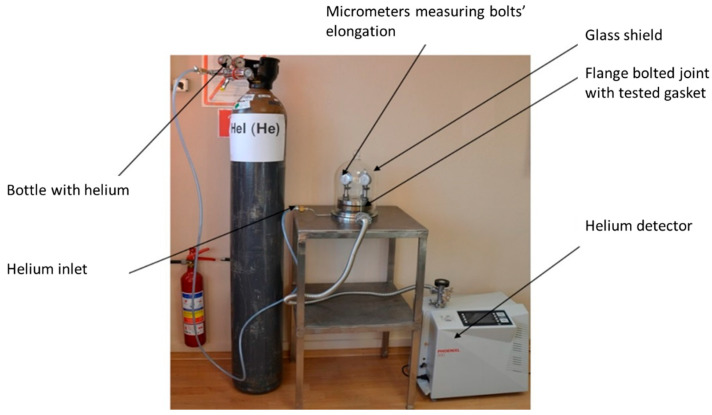
Test stand used in experimental research.

Figure 11 shows the test rig. It consists of two blinded flanges with dimensions corresponding to the DN40 PN40 designation, four M16 bolt connections, micrometres (to measure bolt deformation during tensioning), a helium detector, and a bottle of helium.

The measurement of bolt elongation was based on the method given in DIN 28090-3 [53,54]. It consists of measuring the distance between the face of the bolt shank and the face of the rod embedded in the bolt. Based on the previously determined stiffness characteristics of each bolt (in a tensile test on a testing machine), the relationship between the tension force of the bolt and its elongation was known. Two edge gasket solutions, geometrically identical to the models shown in Figure 1, were used in the tests.

The objective of the research was to determine the leakage as a function of the tension force of a single bolt. Bolt tension was induced with a torque spanner by tracking the elongation of a given bolt on a micrometre sensor. Bolt tension values ranged from 5 kN to 25 kN with increments of 5 kN. Once the appropriate bolt tension was induced, the joint was sealed in a glass dome. Subsequently, a pressure value of 4 MPa was set on the helium bottle pressure-reducing valve and the medium was introduced inside the bolted flange joint. At each set bolt tension value, the tightness of the joint was measured using a spectrometric leak detector [55].

## 3. Results

### 3.1. Results of Numerical Calculations

**Figure 12:**
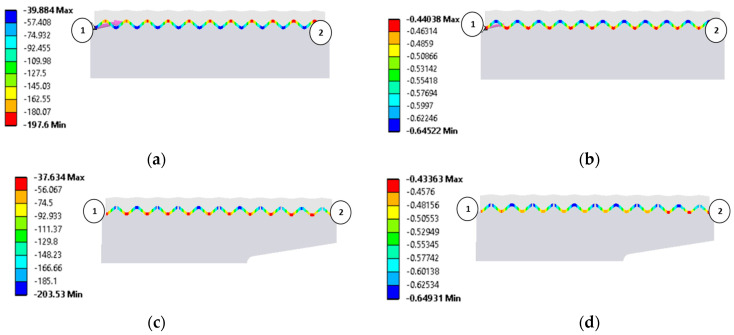
Results of numerical calculations: (**a**) contact pressure between the graphite layer and the ridges of the symmetric gasket core (MPa); (**b**) strain of graphite layer for the symmetric gasket core (mm); (**c**) contact pressure between the graphite layer and the ridges of the asymmetric gasket core (MPa); (**d**) strain of graphite layer for the asymmetric gasket core (mm).

Figure 12 shows the distribution of contact pressure and strain between the graphite sealing layer at the particular ridges of the metal core of the gasket.

The pressure was measured along a measurement path defined in the direction from the inner diameter of the gasket (marked in Figure 12a,b by point 1) to the outer diameter of the gasket (marked in Figure 12a,b by point 2). Figure 12a,b show the pressure and strain distributions in the gasket with the symmetric core, while Figure 12c,d show the pressure and strain distributions in the gasket with the asymmetric core.

**Figure 13:**
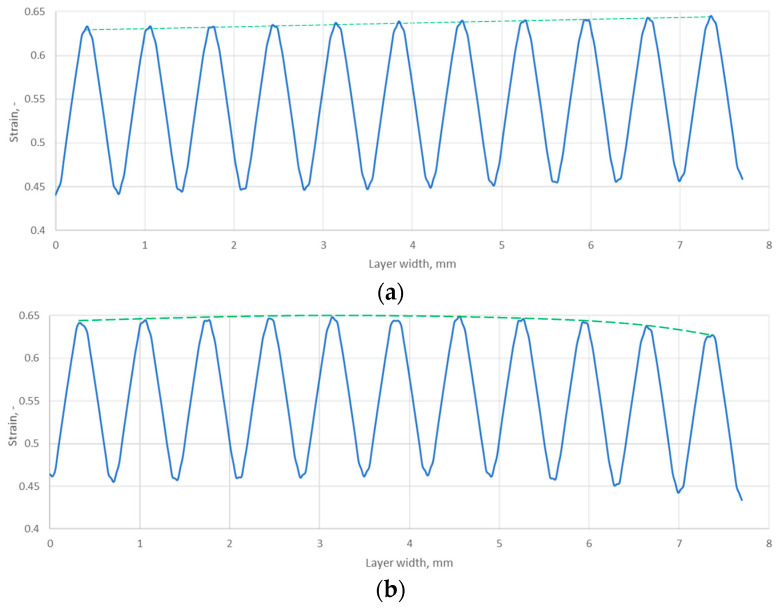
Strain distribution of the graphite layer along the width of the gasket for (**a**) symmetric core and (**b**) asymmetric core.

To better illustrate the effect of the core shape on the graphite layer strain, its progression is shown in the form of graphs in Figure 13. It can be seen that the symmetric core induces a gradual increase in the graphite layer strain as the outer diameter of the gasket is approached. In the case of the gasket with the asymmetric core shape, the highest layer strain has moved to the inner diameter region and maintains an almost constant value up to about 80% of the gasket width. The decrease in strain of the graphite layer drops only at the last three ridges of the core.

### 3.2. Results of Analytical Calculations

**Figure 14:**
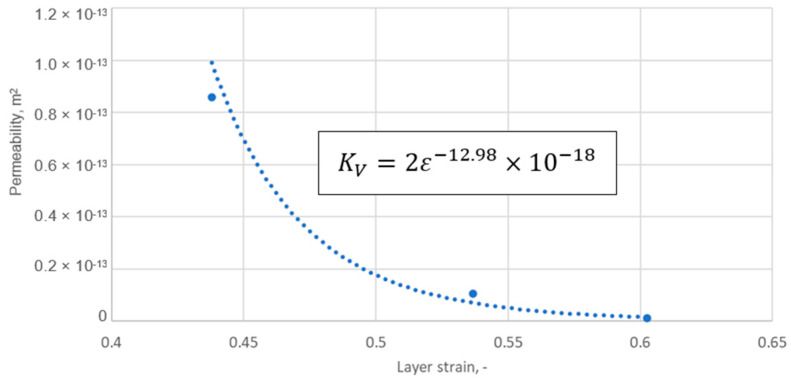
Permeability of the graphite layer as a function of layer strain.

Table 3 presents a summary of the results of the analytical calculations in the form of the average strain of the graphite layer, the permeability, and the radii characterising the position of a given ridge pile-up zone (determined by the inner and outer diameters of a given ridge cut on the core). It should be noted that the average strain values of the graphite layer were calculated from the results of the numerical calculations as the arithmetic mean value from the pile-up at each ridge (for the data in Figure 13). In turn, the permeability value in each zone was counted as a function of the arithmetic mean strain of the zone. The dependence of the permeability relationship as a function of strain is shown in Figure 14. The fit of the regression curve to the obtained characteristic points was reached with a determination coefficient R^2^ of 0.998. The effect of the uniformity of the strain of the graphite layer, and the transfer of its maximum value to the inner diameter area of the gasket, is very beneficial. As shown in [34], the concentration of contact pressure and sealing layer strain is closer to the inner diameter, the sealing tightness increases.

The equation describing permeability as a function of strain was defined as follows:

(7) $$K_{V}=2\varepsilon^{-12.98}{\times 10}^{-18}$$

**Figure 15:**
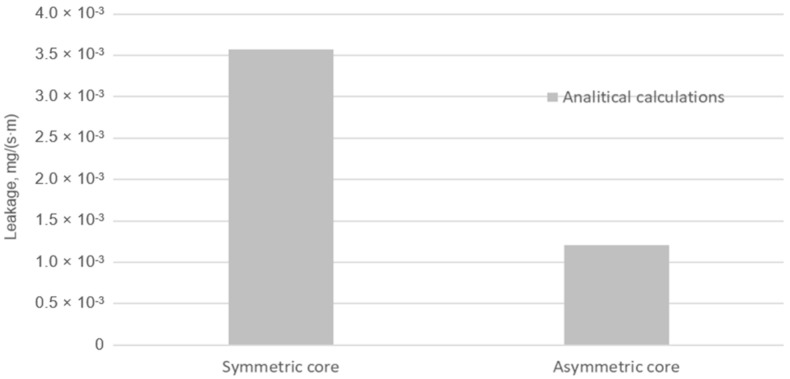
Leakage from the symmetrical and asymmetrical core gasket.

Based on Equations (6) and (7), the fluid leakage through the graphite sealing layer was calculated at the maximum applied tension of a single bolt of 25 kN. The calculated leakage values are shown in Figure 15. For the gasket with the symmetrical core, the leakage is almost three times higher. The analytically calculated leakage was 3.57 × 10^−3^ mg/(s·m) and 1.21 × 10^−3^ mg/(s·m) for the symmetrical and asymmetrical core cases, respectively.

### 3.3. Results of Experimental Measurements

**Figure 16:**
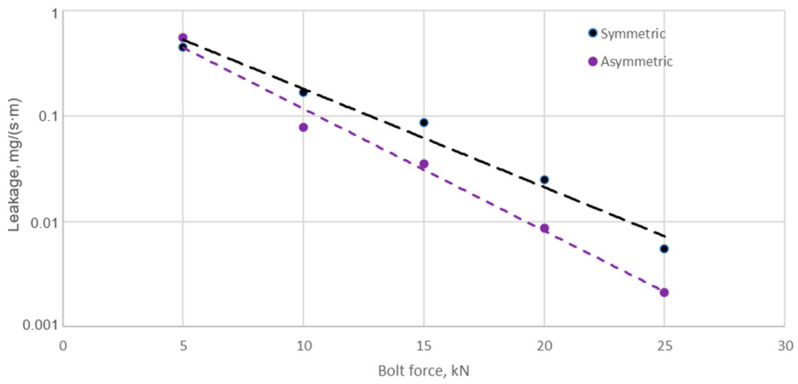
Leakage from the symmetrical and asymmetrical core gasket as a function of single bolt tension.

Figure 16 illustrates the leakage values measured experimentally at successive joint-tensioning steps.

**Figure 17:**
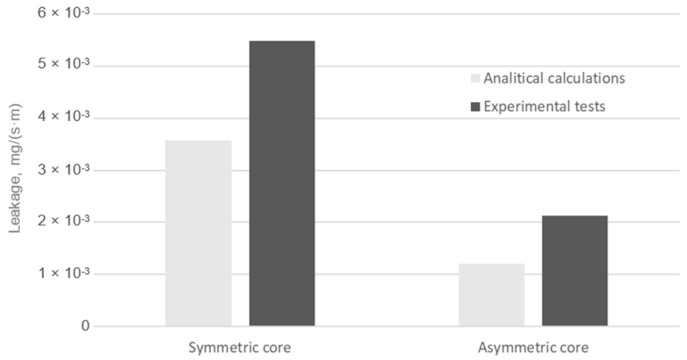
Leakage from the symmetrical and asymmetrical core gasket according to experimental tests and analytical calculations.

From the data shown in Figure 16, it can be seen that increasing the bolt tension of the joint leads to a progressively faster increase in leakage from the gasket with the asymmetric core compared to the gasket with the symmetric core. At a single bolt tension of 25 kN, the leakage from the symmetric core gasket is 5.48 × 10^−3^ mg/(s·m), while the leakage from the asymmetric core gasket is 2.12 × 10^−3^ mg/(s·m). This represents more than a 60% increase in the leakage of the gasket with the asymmetric core compared to the gasket with the symmetric core. Figure 17 summarises the leakage values measured experimentally against those obtained analytically (at a maximum bolt tension of 25 kN).

The percentage error between the mean value of the leakage measured experimentally and that obtained from analytical calculations is 34% and 43% for the symmetric core gasket and the asymmetric core gasket, respectively. The error between the measured and analytically calculated leakage is relatively large. This is a direct result of the measurement uncertainty of the detector used on the test stand, which can be as high as 30%. These differences may indicate a further need to expand the analytical model. On the other hand, both analytical calculations and measurements showed a higher sealing capacity for a gasket with an asymmetric core compared to a gasket with a symmetric core.

## 4. Discussion

### 4.1. Explanation of the Leakage Mechanism

The circumferential notch on the outer edge of the gasket core positively increased the strain in the graphite layer at the individual core ridges and resulted in a more uniform strain distribution of this layer along the width of the gasket. At the maximum tension force of a single bolt (i.e., 25 kN), the average strain value on ridges 1 to 9 is approximately 0.551. Only on the last two ridges (10 and 11) was there a slight decrease in strain to an average value of 0.535. In the case of a gasket with a symmetric core, the strain distribution of the graphite layer on the individual ridges is uneven. The smallest strain value (i.e., 0.514) occurs on the side of the inner gasket diameter and gradually increases towards the outer diameter, reaching a maximum strain value of 0.538. Only at the last two ridges does the strain of the graphite layer in the symmetric core approach values that are nearly identical to those occurring over the entire width range of the gasket with an asymmetric core. The increase in the strain of the graphite layer results in greater compaction of the pores in its internal structure and a more effective filling of the irregularities of the sealed metal surfaces, resulting in a reduction in leakage.

In general, the sealing mechanism at the interface of two surfaces separated by a sealing material should be considered in terms of two leakage components: the leakage component at the interface of the connected surfaces and the leakage component through the sealing material itself. In this case, the leakage model can be seen as a combination of two porous layers (in the form of rings) with different permeabilities and thicknesses. Consequently, the total leakage can be described by the following general form:

(8) $$Q=Q\prime f\left(K_{1}\right)+Q\prime \prime f(K_{2})$$

**Figure 18:**
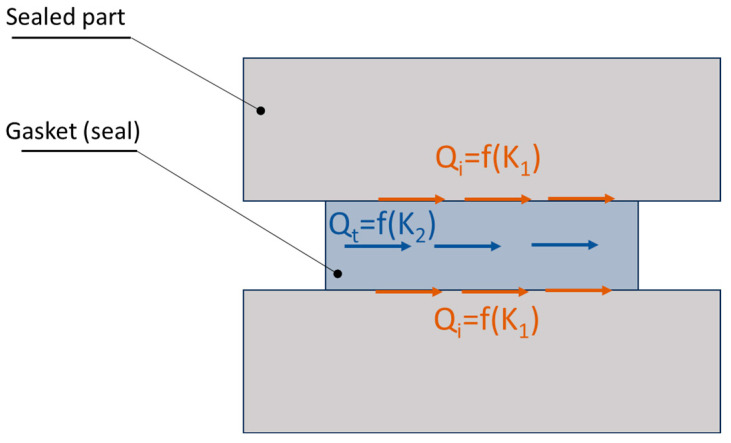
Potential leakage paths in the area of contact between two surfaces separated by sealing material.

The phenomenon of total leakage is also illustrated in Figure 18.

The leakage component related to the permeability at interface *K*_1_ depends mainly on the surface roughness. The second component *K*_2_ depends mainly on the porosity of the sealing material. In both cases, the modelling of the leakage is reduced to the determination of the permeability of the porous layer *K*. The permeability *K*_1_ can be specified by means of a fractal description of the surface roughness profile at the interface, as presented in [56,57]. The result of this approach is the determination of the permeability in the following form:

(9) $$K_{1}=\frac{\pi D_{f}\lambda_{max}^{4}}{128\tau \left(4-D_{f}\right)S}$$

The main parameter of this model is the maximum pore diameter that forms between the contact of a flat surface and a rough surface and is related to the maximum peak of the roughness profile:

(10) $$\lambda_{max}=\frac{S_{max,p}+\xi l_{max,p}}{P_{max,p}+2\xi +l_{max,p}}$$

This diameter can be determined by measuring the roughness profile and identifying the constants *G* and *D*, which define the circumference and area of the maximum peak of the roughness profile.

Another parameter is capillary tortuosity, calculated from the following relationship:

(11) $$\tau =\frac{1}{2}\left[1+\frac{1}{2}\sqrt{1-\epsilon }+\sqrt{1-\epsilon }\frac{\sqrt{{\left(\frac{1}{\sqrt{1-\epsilon }}-1\right)}^{2}+\frac{1}{4}}}{1-\sqrt{1-\epsilon }}\right]$$

Meanwhile, *ϵ* denotes the porosity of the bed, expressed in the following relationship:

(12) $$\epsilon =\frac{V_{t,p1}+V_{t,p2}}{L_{x}L_{y}h}$$

where *V_t,p_*_1_ and *V_t,p_*_2_ denote the pore volume above and below the mean line of the roughness profile (the reference plane), respectively; *L_x_L_y_h* indicates the product denoting the unit volume of the porous layer at the interface.

Another parameter that significantly affects the tightness is the fractal dimension of the capillary tortuosity, which is calculated from the following relationship:

(13) $$D_{f}=2-\frac{ln\epsilon }{ln(\lambda_{min}/\lambda_{max})}$$

In the above model, the height of the porous layer can be calculated from the degree of penetration of the smooth layer into the rough one, using the fundamental laws of contact mechanics. Based on these laws, three basic deformation regimes are distinguished: elastic, elastoplastic, and plastic [56].

The permeability through the material can, in turn, be described using the model proposed in the following [58]:

(14) $$K_{2}=\frac{\pi D_{f}{\left(\frac{\lambda_{max}}{2}\right)}^{D_{f}}\left({\frac{\lambda_{max}}{2}}^{3-D_{f}+D_{t}}-{\frac{\lambda_{min}}{2}}^{3-D_{f}+D_{t}}\right)}{2^{4-D_{t}}\sqrt{A^{1+D_{t}}}\left(3-D_{f}+D_{t}\right)}$$

where *D_t_* and *A* denote the fractal dimension of capillary tortuosity and the cross-sectional area of the representative element, respectively, calculated from the following relationships:

(15) $$D_{t}=\left(2-D_{f}+1\right)+\left(2-D_{f}\right)\frac{ln{\;D}_{f}-ln\left(D_{f}-1\right)}{ln\epsilon }$$

(16) $$A=\frac{\pi D_{f}{\left(\frac{\lambda_{max}}{2}\right)}^{D_{f}}\left({\frac{\lambda_{max}}{2}}^{2-D_{f}}-{\frac{\lambda_{min}}{2}}^{2-D_{f}}\right)}{\epsilon \left(2-D_{f}\right)}$$

The dominance of one component of the leakage over the other depends on the type of gasket used and, more specifically, on the material from which the gasket is made.

**Figure 19:**
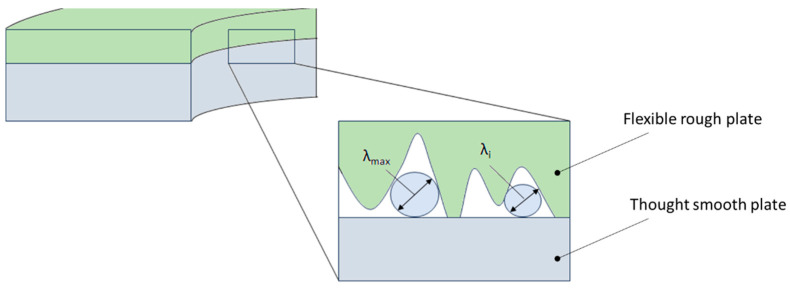
Potential leakage path at the metal–metal interface.

**Figure 20:**
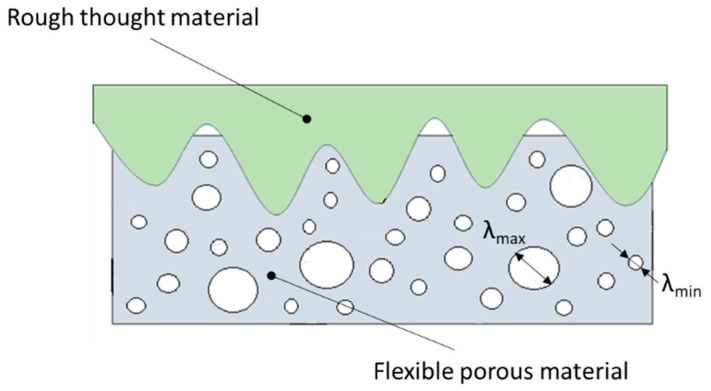
Potential leakage path at the metal–soft sealing material interface with a high porosity structure.

In the case of a metal gasket, where the porosity of the structure is several orders of magnitude lower compared to a softer gasket material (e.g., made of expanded graphite), the sealing mechanism associated with the component at the interface of the connected surfaces dominates. In the case of a highly porous gasket, the component related to the leakage mechanism through the material itself dominates. Even with a small strain, the soft material very effectively fills the irregularities of the rough metal surface, so the main leakage occurs through the porous material structure. The differences between these two sealing mechanisms are shown in Figure 19 and Figure 20.

The multi-edge gasket presented in this paper combines both sealing mechanisms. Due to the high porosity of the graphite layer and the high deformability of the material, it effectively fills the irregularities of the metal surfaces, both at the interface of the joined flanges and at the interface with the metal core. Based on the sealing mechanism outlined above, it can be concluded that leakage through the graphite layer is the dominant component of leakage.

This can be evidenced through the research results reported in [59], in which the porosity and pore size distribution in the graphite layer were determined as a function of the contact pressure exerted on it—see Table 4.

**Figure 21:**
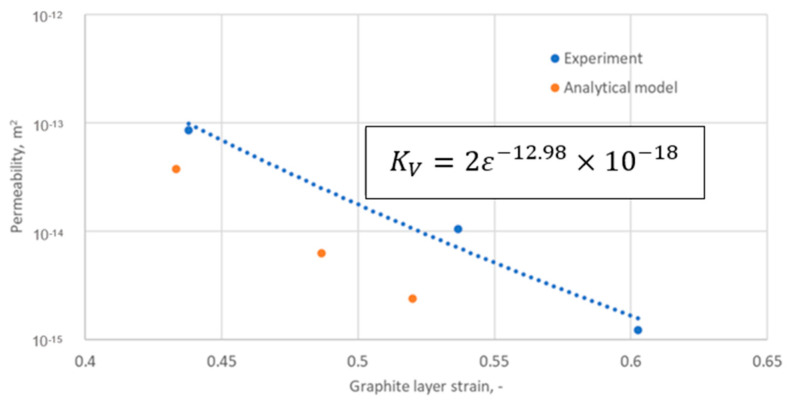
Permeability of the graphite layer as a function of graphite layer strain.

In Figure 21, the permeability values calculated using Equation (14) (based on data from Table 3) are compared with the experimentally determined permeability values. The values calculated by the model, compared to the measured ones, can be considered satisfactory. It can be seen that, in the strain range from 0.43 to 0.52, the permeability calculated through the model varies from 3.8 × 10^−14^ to 2.4 × 10^−15^, while the experimentally determined permeability in the comparable strain range, i.e., from 0.44 to 0.53, varies from 8.6 × 10^−14^ to 1.2 × 10^−15^.

### 4.2. Effect of Gasket Width on Leakage

The outer notch in the core caused a significant increase in the average strain of the graphite layer. An increase in this strain (at the same tension force of a single bolt) can be achieved by reducing the gasket width. However, reducing the gasket width leads to increased leakage. The effect of decreasing the gasket width on leakage is well illustrated in the following equations:

(17) Q=2πKC

(18) $$C=\frac{\left(1-\varepsilon \right)}{ln\beta }$$

where *C* denotes the coefficient linking the effect of strain and the relative width *β* of the ring on leakage.

**Figure 22:**
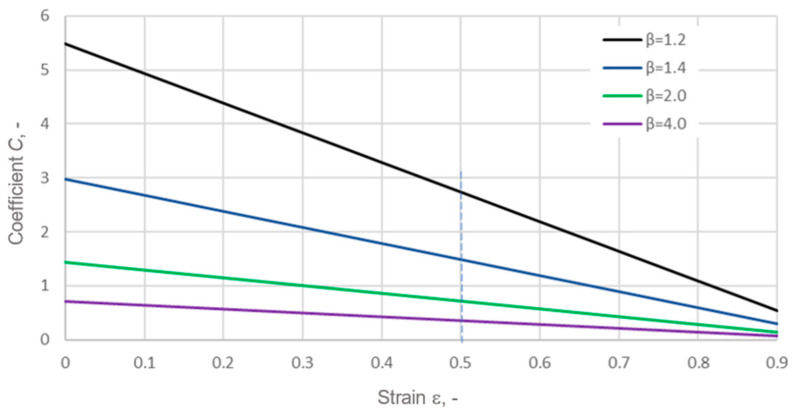
Effect of strain and the relative width of the ring on the coefficient, *C*.

The relative width, *β*, of the ring is expressed as the ratio of its outer radius to the inner radius. The effect of strain and the relative width, *β*, of the ring on the coefficient, *C*, is shown in Figure 22.

It can be seen that, for a relatively small gasket width (i.e., for *β* = 2), a reduction in the gasket width leads to a multiple increase in the coefficient, C, which directly translates into an increase in leakage. Therefore, the design of a gasket with an asymmetric core allows the strain of the graphite layer to be increased without reducing the gasket width. Increasing the strain of the porous graphite layer results in greater compaction of the layer, which, in turn, reduces its permeability and leads to a higher degree of filling of the roughness profile of the surfaces to be sealed, quite like in the case of bonded surfaces [60,61].

## 5. Conclusions

Based on the performed calculations, the experimental studies, and the results obtained, the following conclusions can be drawn:

1. An increase in the strain of the graphite layer and its uniform distribution over the individual locking ridges can be achieved by notching the outer diameter of the metal core of the gasket. This leads to an extension of the strain and its uniformity across the width of the graphite layer. The average strain value of the graphite layer with the notched core (at the maximum joint tension force, i.e., 25 kN per bolt) rose by approximately 7% compared to the gasket with a symmetric core.
2. The increase in the average strain value of the graphite layer on the individual ridges in the gasket with the notched core resulted in an increase in tightness by approximately 60% compared to the gasket with the symmetric, non-notched core.
3. The greater the strain of the graphite layer, the better it fills the surface irregularities at the interface between the graphite layer and the metal flange surface, as well as at the interface between the graphite layer and the metal core of the gasket. Additionally, this results in greater compaction of the graphite material and a reduction in its porosity, which leads to a reduction in leakage.
4. Two distinctive sealing mechanisms can be distinguished in the considered design of a multi-edge gasket with expanded graphite inserts. The first occurs at the interface between the graphite layer and the metal surfaces, while the second takes place in the internal structure of the graphite layer. The level of tightness at the metal–graphite interface depends primarily on the height of the metal surface irregularities and the compressibility of the graphite. The level of tightness in the graphite layer depends mainly on its degree of compaction.
5. Due to the high elasticity of the graphite layer, there is an almost complete filling of the surface irregularities of the metal flanges at the interface with the metal core of the gasket. This means that leakage through the graphite material is the dominant component of leakage. This is evidenced by the results of analytical permeability calculations for the graphite layer, compared with the results obtained experimentally. With a strain of the graphite layer in the range of 0.42 to 0.52, the calculated and experimentally measured permeability values fall within the same order of magnitude, i.e., from 1 × 10^−14^ to 1 × 10^−15^, which, with such low values, makes the accuracy of the obtained results satisfactory.
6. At the maximum tension force (i.e., 25 kN per bolt), a friction coefficient at the graphite–metal interface in the range from 0.1 to 0.3 does not significantly affect the numerical calculations of the strain distribution in the graphite layer.
7. The paper considers only one type of asymmetry related to the circumferential notch on the outer diameter of the gasket. In order to address this limitation, the next step would be to investigate how changes in notch depth, position, or modification of the inner edge can affect the leakage behaviour.
8. In further phases of the research, it is planned to extend the test stand to allow leakage measurements at elevated temperatures.
9. It should also be noted as important to investigate larger gasket sizes. This is because, as the diameter increases, the width of the gasket also increases, which generally leads to greater variability in the contact pressure on the sealing surface.

## Figures and Tables

**Table 1:** Material properties of the metal parts of the joint.

Part	Material	Young’s Modulus, GPa	Compression Strength, MPa	Poisson’s Ratio, –
Flange	304L (1.4307)	197	115	0.3
Gasket core	316L (1.4404)	200	220	0.3

**Table 2:** Contact conditions at the graphite-metal interface.

Type of Contact	Frictional
Friction value	From 0.1 to 0.3 with an increment of 0.05
Contact behaviour	Symmetric
Formulation	Augmented Lagrange
Detection method	On Gauss point
Penetration tolerance	0.01 mm
Normal stiffness factor	1

**Table 3:** Partial strain and permeability values at individual ridges of the gasket core.

Core ridge No. 1			Core ridge No. 2		
Parameters	Symmetric core	Asymmetric core	Parameters	Symmetric core	Asymmetric core
*ε*, –	0.514	0.547	*ε*, –	0.515	0.548
*K_V_*, m^2^	6.846 × 10^−15^	4.999 × 10^−15^	*K_V_*, m^2^	6.665 × 10^−15^	4.903 × 10^−15^
*r*_1_, mm	26.65	26.65	*r*_2_, mm	27.35	27.35
*r*_2_, mm	27.35	27.35	*r*_3_, mm	28.05	28.05
Core ridge No. 3			Core ridge No. 4		
Parameters	Symmetric core	Asymmetric core	Parameters	Symmetric core	Asymmetric core
*ε*, –	0.520	0.553	*ε*, –	0.522	0.555
*K_V_*, m^2^	5.914 × 10^−15^	4.345 × 10^−15^	*K_V_*, m^2^	5.721 × 10^−15^	4.201 × 10^−15^
*r*_3_, mm	28.05	28.05	*r*_4_, mm	28.75	28.75
*r*_4_, mm	28.75	28.75	*r*_5_, mm	29.45	29.45
Core ridge No. 5			Core ridge No. 6		
Parameters	Symmetric core	Asymmetric core	Parameters	Symmetric core	Asymmetric core
*ε*, –	0.529	0.552	*ε*, –	0.521	0.553
*K_V_*, m^2^	6.053 × 10^−15^	4.449 × 10^−15^	*K_V_*, m^2^	5.847 × 10^−15^	4.356 × 10^−15^
*r*_5_, mm	29.45	29.45	*r*_6_, mm	30.15	30.15
*r*_6_, mm	30.15	30.15	*r*_7_, mm	30.85	30.85
Core ridge No. 7			Core ridge No. 8		
Parameters	Symmetric core	Asymmetric core	Parameters	Symmetric core	Asymmetric core
*ε*, –	0.522	0.553	*ε*, –	0.524	0.551
*K_V_*, m^2^	5.630 × 10^−15^	4.348 × 10^−15^	*K_V_*, m^2^	5.415 × 10^−15^	4.632 × 10^−15^
*r*_7_, mm	30.85	30.85	*r*_8_, mm	31.55	31.55
*r*_8_, mm	31.55	31.55	*r*_9_, mm	32.25	32.25
Core ridge No. 9			Core ridge No. 10		
Parameters	Symmetric core	Asymmetric core	Parameters	Symmetric core	Asymmetric core
*ε*, –	0.533	0.554	*ε*, –	0.537	0.539
*K_V_*, m^2^	4.343 × 10^−15^	4.317 × 10^−15^	*K_V_*, m^2^	5.022 × 10^−15^	6.044 × 10^−15^
*r*_9_, mm	32.25	32.25	*r*_10_, mm	32.94	32.94
*r*_10_, mm	32.94	32.94	*r*_11_, mm	33.64	33.64
Core ridge No. 11					
Parameters	Symmetric core	Asymmetric core			
*ε*, –	0.539	0.532			
*K_V_*, m^2^	4.821 × 10^−15^	7.201 × 10^−15^			
*r*_11_, mm	33.64	33.64			
*r*_12_, mm	34.35	34.35			

**Table 4:** Selected properties of the graphite layer as a function of contact pressure [59].

Contact Pressure, MPa	Thickness, mm	Strain, -	Porosity, -	Max. Pore Diameter, mm	Min. Pore Diameter, mm
0	1.50	–	0.558	–	–
20	0.85	0.43	0.217	3.03 × 10^−3^	0.54 × 10^−3^
40	0.77	0.49	0.142	1.81 × 10^−3^	0.20 × 10^−3^
100	0.72	0.52	0.075	1.32 × 10^−3^	0.09 × 10^−3^

## Data Availability

The original contributions presented in the study are included in the article, further inquiries can be directed to the corresponding authors.
